# Proteomic Analysis of *Trypanosoma cruzi* Response to Ionizing Radiation Stress

**DOI:** 10.1371/journal.pone.0097526

**Published:** 2014-05-19

**Authors:** Helaine Graziele Santos Vieira, Priscila Grynberg, Mainá Bitar, Simone da Fonseca Pires, Heron Oliveira Hilário, Andrea Mara Macedo, Carlos Renato Machado, Hélida Monteiro de Andrade, Glória Regina Franco

**Affiliations:** 1 Departamento de Bioquímica e Imunologia, Universidade Federal de Minas Gerais, Belo Horizonte, Minas Gerais, Brazil; 2 Departamento de Parasitologia, Universidade Federal de Minas Gerais, Belo Horizonte, Minas Gerais, Brazil; 3 Embrapa Recursos Genéticos e Biotecnologia, Brasília, Distrito Federal, Brazil; ENEA, Italy

## Abstract

*Trypanosoma cruzi*, the causative agent of Chagas disease, is extremely resistant to ionizing radiation, enduring up to 1.5 kGy of gamma rays. Ionizing radiation can damage the DNA molecule both directly, resulting in double-strand breaks, and indirectly, as a consequence of reactive oxygen species production. After a dose of 500 Gy of gamma rays, the parasite genome is fragmented, but the chromosomal bands are restored within 48 hours. Under such conditions, cell growth arrests for up to 120 hours and the parasites resume normal growth after this period. To better understand the parasite response to ionizing radiation, we analyzed the proteome of irradiated (4, 24, and 96 hours after irradiation) and non-irradiated *T. cruzi* using two-dimensional differential gel electrophoresis followed by mass spectrometry for protein identification. A total of 543 spots were found to be differentially expressed, from which 215 were identified. These identified protein spots represent different isoforms of only 53 proteins. We observed a tendency for overexpression of proteins with molecular weights below predicted, indicating that these may be processed, yielding shorter polypeptides. The presence of shorter protein isoforms after irradiation suggests the occurrence of post-translational modifications and/or processing in response to gamma radiation stress. Our results also indicate that active translation is essential for the recovery of parasites from ionizing radiation damage. This study therefore reveals the peculiar response of *T. cruzi* to ionizing radiation, raising questions about how this organism can change its protein expression to survive such a harmful stress.

## Introduction

Chagas disease, a neglected tropical disease caused by the protozoan parasite *Trypanosoma cruzi*, is considered to be a public health problem [1], [2]. Over 10 million people are infected in Latin America and more than 100 million individuals live at risk of infection by blood transfusion, congenital, or oral transmission [3]. Forty years after its introduction, benznidazole and nifurtimox continue to be the first choice of treatment for Chagas disease. However, chemotherapy based on nitroheterocyclic compounds has a limited efficacy for patients in the chronic phase of infection and these drugs are highly toxic [4],[5]. Little progress has been made toward the treatment of infected individuals and the development of more efficient drugs to treat Chagas disease patients remains urgent. Considering the resistance of some parasites to chemotherapy, the introduction of vaccines against *T. cruzi* could be another option [3], [6].

*T. cruzi* is capable of resisting high doses of gamma radiation, enduring up to 1.5 kGy. As a direct biological effect, gamma radiation causes double-strand breaks (DSB) in the parasite DNA. However, 48 hours after irradiation, it is possible to see the chromosomal bands already restored. The parasite growth arrests for up to 120 hours, returning to the normal rate after this period [7], [8]. This extraordinary recovery might be due to a very efficient DNA repair system. Homologous recombination is required to repair DNA DSBs and the involvement of the TcRAD51 protein in this process was evaluated by our group elsewhere. The overexpression of TcRAD51 ensures a more effective DSB DNA repair and a greater resistance to DNA damage in *T. cruzi* [9].

Oxidative stress is another effect of ionizing radiation due to the production of hydroxyl radicals (OH^•^), superoxide (O_2_^•^), and hydrogen peroxide (H_2_O_2_), directly from radiolysis of water. These products are commonly called reactive oxygen species (ROS) [10]. Once the DNA molecule is intimately associated with water, the production of OH^•^ results in damages that include, apart from DSBs, oxidation of nitrogenous bases and sugar [11], [12]. Approximately 75–80% of the biological damage caused by this type of radiation is mediated by OH^•^ formation. Such radicals are capable of reacting with most biologically relevant molecules. Each amino acid reacts differently with OH^•^ and the precise mechanisms of reaction are poorly understood [13].

Another organism that is extremely resistant to ionizing radiation is the bacterium *Deinococcus radiodurans*, which can withstand radiation doses of up to 15 kGy [14]. *D. radiodurans* presents a very robust DNA repair apparatus; nevertheless, the biological responses to genomic lesions depend on its proteome integrity. Considering that ionizing radiation also induces protein damage through oxidative stress, a protected functional proteome ensures an efficient cell recovery from this type of stress [15]. Using the classical proteomic approach of two-dimensional differential gel electrophoresis (2D-DIGE) coupled with mass spectrometry (MS), Basu & Apte observed in a time-course analysis that some classes of proteins have a strong influence on stress responses. These proteins are mainly involved in processes such as DNA damage repair, protein synthesis and folding, and responses to oxidative stress [16].

Proteome *versus* transcriptome analyses have been highly recommended for studies with tripanosomatids, as they have very peculiar molecular features concerning their gene expression control. As a kinetoplastid, *T. cruzi* transcription is polycistronic and gene regulation occurs mainly post-transcriptionally, with mature mRNAs being generated by trans-splicing and polyadenylation [17], [18]. The processing and stabilization of mRNAs are extremely important in trypanosomatid gene regulation [19], [20]. Furthermore, other dynamic control mechanisms, such as post-translational modifications, are fundamental in the regulation of gene expression and need to be better characterized in these organisms [21]–[23].

A time-course microarray study previously carried out by our group analyzed the *T. cruzi* gene expression in response to gamma radiation [7]. Among the 273 differentially expressed genes, 160 were upregulated and 113 were downregulated. The majority of the genes with assigned functions was downregulated. Translation, protein metabolic processes, and the generation of precursor metabolites and energy pathways were affected. Four mitochondrial genes and Retrotransposon Hot Spot genes were upregulated; likewise, the tyrosyl-DNA phosphodiesterase 1, a gene involved in DNA DSB repair, was also induced [7]. Taking into account the *T. cruzi* gene expression peculiarities, analyses of proteome changes after irradiation in different time points may contribute to the understanding of the parasite response to such stress.

In this work, we performed quantitative proteomic analyses using 2D-DIGE to ascertain the parasite response to ionizing irradiation. A total of 543 protein spots were found to be differentially expressed considering all analyzed time points and 53 different proteins were identified by tandem mass spectrometry (MS/MS). The great majority of the identified proteins was represented by several isoforms, suggesting that post-transcriptional and/or post-translational modifications are occurring as a consequence of gamma radiation exposure. Overexpression of tryparedoxin after irradiation was also observed, indicating that the parasite may be responding to the oxidative stress caused by irradiation. We also compared the time-course microarray and proteomic analyses. Although some of the protein expression patterns confirmed the microarray results, the correlation between mRNA and protein levels of the genes identified in both studies was extremely poor. In addition, treatment of the parasites with translation inhibitors showed that the synthesis of proteins putatively involved in the parasite response to stress is essential for its recovery from such a harmful stress.

## Materials and Methods

### Cell Culture and Gamma Irradiation

In this work, we used *T. cruzi* epimastigote forms of the CL Brener strain, which were isolated and characterized by Brener & Chiari [24]. Clones have been maintained as frozen stocks at Universidade Federal de Minas Gerais. Parasites were grown at 28°C in liver infusion tryptose (LIT) medium pH 7.3, supplemented with 10% fetal bovine serum, streptomycin sulfate (0.2 g/L), and penicillin (200,000 units/L). Cultures in the exponential growth phase (2×10^7^ cells/mL) were exposed for 20 minutes to 500 Gy of gamma radiation (1,578 Gy/h) in a cobalt (60 Co) irradiator (Centro de Desenvolvimento da Tecnologia Nuclear – CDTN, Belo Horizonte, Brazil). Cells were counted daily after irradiation to generate the growth curve.

### Cycloheximide and Puromycin Treatments

Parasites exposed or not exposed to 500 Gy of gamma radiation were treated with cycloheximide (Calbiochem) 50 µg/mL for 15 minutes or with puromycin (Sigma) 25 µg/mL for 1 hour. Both drugs were added to the parasite cultures 4 hours after irradiation. Parasites were washed twice in phosphate buffered saline (137 mM NaCl, 4 mM Na_2_HPO_4_, 1.7 mM KH_2_PO_4_, and 2.7 mM KCl), the LIT medium was replaced, and the cells were counted.

### Protein Extract Preparation and DIGE Labeling

Protein extracts were obtained, simultaneously, in triplicate for each condition: non-irradiated control (NI), 4, 24, and 96 hours after irradiation. Parasites (2×10^9^ cells) were washed twice with LIT medium followed by centrifugation at 1,500 g for 5 minutes at 4°C. Each pellet was resuspended in 200 µL of lysis buffer (8 M urea, 2 M thiourea, 4% CHAPS, 10 mM Tris base) and a protease inhibitor mix (GE Healthcare, USA). Samples were mixed on vortex every 30 minutes during 2 hours of incubation at room temperature and subsequently centrifuged at 14,000 g for 30 minutes. The supernatants were aliquoted and stored at −70°C for further use. For all samples, protein concentration was determined using the 2D Quant kit (GE Healthcare, USA), according to manufacturer's instructions.

Before labeling, samples had their pH adjusted to 8.5 with NaOH 0.05 M (as recommended by the manufacturer's protocol). To reduce biological variation, a pool of protein extracts obtained from triplicates was used. A total of 50 µg of protein from each pool (NI, 4, 24, and 96 hours after irradiation) was labeled with CyDye DIGE Fluor Minimal Labeling Kit (GE Healthcare, USA). The dye swap strategy was used to avoid label bias, where each sample was labeled with 400 pmol of either Cy3 or Cy5. A mixture of all protein extracts (12.5 µg of each pool sample) was labeled with Cy2 as the internal control. Reactions were carried out on ice for 30 minutes in the dark and then stopped by the addition of 10 mM lysine.

### Two-Dimensional Gel Electrophoresis

#### First dimension

The isoelectric focusing (IEF) was performed using Immobiline Dry Strips (GE Healthcare, USA) 18 cm in size, with a pH ranging from 4–7. Strips were loaded with 50 µg of protein per CyDye (total of 150 µg) and sample buffer containing 8 M urea, 2 M thiourea, 4% CHAPS, 1% dithiothreitol (DTT), 0.002% bromophenol blue, and 1% IPG buffer (pH 4–7; GE Healthcare, USA). Passive rehydration followed overnight, at room temperature, in a strip holder (GE Healthcare, USA). The IEF protocol used in the Ettan IPGphor3 (GE Healthcare, USA) instrument was as follows: 50 µA per strip, 20°C, steps 1 to 5: 0.2 kV for 12 hours, 0.5 kV for 2 hours; 1 kV for 1.5 hour, 8 kV for 2 hours, 8 kV gradually raising to 40 kV, accumulating approximately 60 kV in total. Focused IPG strips were equilibrated for 15 minutes in an equilibration solution (50 mM Tris-HCl pH 8.8, 6 M urea, 30% glycerol, 2% SDS, 0.002% bromophenol blue and 125 mM DTT) and then alkylated for an additional 15 minutes in an equilibration solution containing 13.5 mM iodoacetamide instead of DTT.

#### Second dimension

Equilibrated strips were briefly washed in 1x running buffer (25 mM Tris, 192 mM glycine, and 0.2% SDS) and placed on top of 12% acrylamide/bis-acrylamide gels, overlaid with a 0.5% agarose solution. Protein separation was carried out at 10°C, in an Ettan Dalt Six Electrophoresis System (GE Healthcare, USA), 45 mA per gel, until the dye front reached the bottom of the gel. Labeled proteins in each gel were visualized using the Typhoon FLA 9000 scanner (GE Healthcare, USA) at 100 µM image resolution with excitation/emission wavelengths for Cy3 (532/580 nm), Cy5 (633/670 nm), and Cy2 (488/520 nm). Gel images were uploaded and cropped using Image Loader Software (GE Healthcare, USA), then imported to DeCyder 2D software, version 7.0 (GE Healthcare, USA).

### DIGE Data Analysis

For spot detection, the Differential In-gel Analysis (DIA) module of DeCyder 2D software, version 7.0 (GE Healthcare, USA), was used. The DIA co-detection algorithm exploits the identical spot patterns from multiple samples in the same gel. After the removal of some artifacts from the gels, spot quantification was performed automatically by normalizing the spot volumes against the internal control. The following steps were performed in the Biological Variation Analysis module, which uses images processed in DIA and matches spots across gels. One-way ANOVA and Student's t-test were applied to evaluate differential protein expression levels between the groups of study. Spots classified as significantly differentially expressed were manually inspected. Abnormal spots were excluded from the analysis when necessary and gels were re-matched.

### Trypsin in-Gel Digestion, Mass Spectrometry, and Protein Identification

Differentially expressed protein spots were excised and trypsin in-gel digestion was carried out overnight at 37°C with 20 ng/µL of trypsin (Promega, Sequencing Grade Modified Trypsin, USA), diluted in 25 mM ammonium bicarbonate. After trypsin digestion, peptides were extracted from the gel by washing twice with 30 µL of 50% acetonitrile and 5% formic acid solution and shaking for 15 minutes. Peptides were then concentrated (Eppendorf Concentrator 5301) to 10 µL and desalted using Zip-Tip (C18 resin, P10, Millipore Corporation, USA). Once the peptides were eluted (50% acetonitrile/0.1% trifluoroacetic acid) from columns, 0.5 µL of each sample was mixed with 0.25 µL of a saturated matrix solution [10 mg/mL α-cyano-4-hydroxycinnamic acid (Aldrich, USA) in 50% acetonitrile/0.1% trifluoroacetic acid]. Samples were spotted on the MTP AnchorChip 600/384 (Bruker Daltonics) and let to dry at room temperature. Raw data for the identification of proteins were obtained with the MALDI-TOF-TOF AutoFlex III (Bruker Daltonics, USA) instrument (Laboratório Multiusário de Biomoléculas, Departamento de Bioquímica e Imunologia, UFMG, Brazil) in the positive/reflector mode controlled by FlexControl software. Instrument calibration was achieved by using peptide calibration standard II (Bruker Daltonics) as a reference. Trypsin and keratin contamination peaks were excluded from the peak lists used for data base searching. Each spectrum was produced by accumulating data from 200 consecutive laser shots.

MS/MS spectra were searched against the non-redundant protein sequence database from the National Center for Biotechnology Information (http://www.ncbi.nlm.nih.gov) using the MASCOT software (version 2.1) MS/MS ion search tool (http://www.matrixscience.com). The search parameters were as follows: no restrictions on protein molecular weight, two tryptic miss-cleavages allowed, and variable modifications of methionine (oxidation), cysteine (carbamidomethylation), and pyroglutamate formation at N-terminal glutamine of peptides. The mass tolerance for the peptides in the searches was 0.6 Da for MS spectra and 0.4 Da for MS/MS spectra. Peptides were considered to be identified when the scoring value exceeded the identity or extensive homology threshold value calculated by the MASCOT software (p<0.05).

### Manual Curation and Statistical Analysis

Peptide sequences obtained from MASCOT were aligned to the *T. cruzi* annotated genome using the BLAST tool from TriTrypDB (http://www.tritrypdb.org). Protein annotation was reassigned particularly when partial sequences were chosen by MASCOT and full-length sequences were available at the TriTrypDB. Once a final annotated and curated set of upregulated and downregulated spots was available, it was possible to assess the protein species by their expected and observed weights (retrieved from the TriTrypDB and calculated from the position in the 2D-DIGE, respectively).

Statistical analyses were performed using R in-house scripts with built-in statistical functions. A linear model was applied to test the correlation between molecular weight and fold-change. The Wilcoxon test was used to evaluate the presence of significant differences between 1) the observed molecular weights of upregulated and downregulated protein spots and 2) the observed and expected molecular weights from upregulated and downregulated protein spots.

The final set of proteins was further manually annotated according to biological function and grouped into different functional classes based on literature data describing each protein and its molecular role.

## Results and Discussion

### The Effects of Protein Synthesis Inhibition on the Growth of *T. cruzi* Epimastigote Cells Exposed to Gamma Radiation

**Figure 1:**
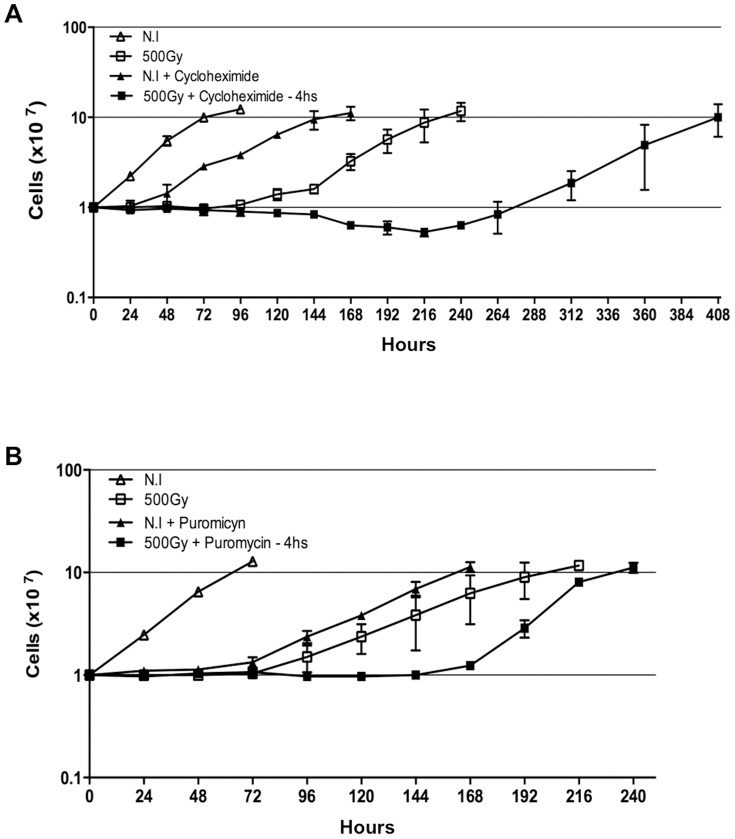
The effect of irradiation and translation inhibition on *T. cruzi* epimastigotes growth. Irradiated (500 Gy) or NI parasites were treated with cycloheximide 50 µg/mL (A) or puromycin 25 µg/mL (B), both added 4 hours after irradiation. Each point represents the mean ± standard deviation of three different experiments.

Normal growth of epimatigote cells was affected by protein synthesis inhibition (using 50 µg/mL cycloheximide or 25 µg/mL puromycin) and by ionizing radiation treatment (500 Gy), as shown in Figure 1. However, irradiation promoted a more drastic growth arrest that persisted for approximately 96 hours; after this period, the parasites resumed normal growth, reaching the stationary phase 216 to 240 hours after irradiation (Figure 1). The treatment of NI cells with cycloheximide (Figure 1A) or puromycin (Figure 1B) retarded the cell growth by at least 24 hours when compared with non-treated cells, but did not lead to parasite death. Conversely, the combination of cycloheximide treatment and gamma radiation was lethal to 40% of the parasites. The remaining parasites resumed growth only 270 hours after irradiation, reaching the stationary phase 408 hours after irradiation (Figure 1A). For puromycin, a similar effect was observed, but treated cells resumed normal growth earlier when compared with cycloheximide-treated parasites (Figure 1B) and, in this case, no parasite death was detected.

### Analysis of the Proteome Profile of *T. cruzi* Epimastigote Cells Exposed to Gamma Radiation

**Figure 2:**
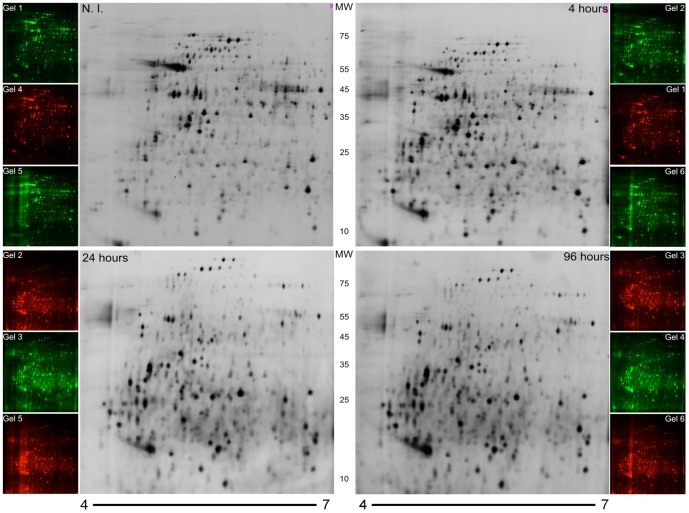
2D-DIGE analysis of total protein extracts of irradiated and NI epimastigote cells. Gel images 1–6 (see the experimental design in Table 1) showing – in triplicate – parasite proteins from each time point, labeled either with Cy3 (green) or Cy5 (red). Proteins were separated in the first dimension along a pH gradient (pH 4–7, 18 cm Immobiline DryStrip (GE Healthcare, USA), and in the second dimension in a 12% polyacrylamide gel. The molecular weight marker (MW) is indicated in kDa.

Since we have verified that newly synthesized proteins have an impact on parasite recovery from irradiation stress, we decided to analyze time-course *T. cruzi* changes in the proteome induced by irradiation. Protein extracts were obtained from control NI cells and 4, 24, and 96 hours after irradiation. No significant losses in the total protein content and integrity were observed by 1D-gel electrophoresis (Figure S1). Using the 2D-DIGE approach, six gels were produced following the experimental design specified in Table 1. This technique was chosen due to its greater sensitivity, reduced gel-to-gel variation, and its capacity for quantitative measurements of the relative abundance of each protein in a complex sample [25]. Figure 2 illustrates 2D-DIGE gels at all time points. An average of 2,186±140 spots was found when compared with the master gel. From those, 543 presented altered expressions after irradiation, considering all time points (one-way ANOVA, p<0.01) and 215 were identified by peptide mass fingerprint, corresponding to 53 different proteins (Table 2). Almost half of these proteins (26) were represented by more than one spot in the 2D gel (ranging from 2–12 spots per protein), indicating the presence of several isoforms for the same protein. These results suggest that post-translational modifications or protein processing are occurring during the response to gamma radiation stress. We have manually annotated the function of all 53 identified proteins via a literature search. Proteins were then manually assigned to 15 different classes according to their biological function (Figure S3).

**Table 1:** Experimental design.

Gel	NI	4 h	24 h	96 h	Pool
1	Cy3	Cy5			Cy2
2		Cy3	Cy5		Cy2
3			Cy3	Cy5	Cy2
4	Cy5			Cy3	Cy2
5	Cy3		Cy5		Cy2
6		Cy3		Cy5	Cy2

Each two-dimensional gel was loaded with 50 µg of total protein extract per sample, labeled either with Cy3 or Cy5. The internal control (a pool containing 50 µg of all time point proteins: NI, 4, 24, and 96 hours after irradiation) was labeled with Cy2.

**Table 2:** Protein data for the 53 proteins identified in this study.

			Observed/Expected		Fold-change				Mascot MS/MS ion search		
Description	TriTrypDB ID (TcCLB.)	Ref. Spot	pI	MW	4 hs	24 hs	96 hs	Anova	Peptides matched	Sequence covarage	Mascot score
14-3-3 protein; putative	511167.90	153	5.00/4.78	24.3/29.1	−1.35	1.08	−1.16	5.61E–5	2	12%	73
40S ribosomal protein S12, putative	508551.20	195	4.82/4.78	10.9/15.9	1.45	3.52	2.92	1.32E–5	2	24%	110
Actin, putative	510571.39 or 510127.79 or 510571.30	66	5.75/5.46	45.9/41.2	−1.64	−14.92	−12.65	8.20E–9	3	10%	136
Alpha tubulin, putative	411235.9	69	6.07/4.7	44.7/49.8	−1.01	−12.65	−10.53	2.00E–7	5	16%	248
		99	5/4.7	55.6/49.8	−1.94	−31.15	−48.82	2.97E–8	11	42%	89
		100	5.06/4.7	55.2/49.8	−2.11	−47.2	−45.08	1.34E–9	5	15%	275
		136	5.29/4.7	29.9/49.8	1.54	−2.02	−1.68	8.44E–9	4	14%	247
		138	5.47/4.7	30.4/49.8	1.09	−2.34	−2.21	8.55E–7	3	9%	155
		139	5.45/4.7	28.9/49.8	1.36	−1.95	−1.63	6.52E–8	4	13%	237
Aminopeptidase, putative,metallo-peptidase, Clan MF, Family M17, putative	508799.240	35	6.22/6.44	58.9/55.9	−1.55	−−1.53	−1.73	5.40E–5	1	2%	52
ATPase beta subunit	509233.180	26	5.15/5.07	58.6/55.7	−2.42	−21.04	−18.44	7.26E–7	5	18%	253
Beta tubulin, putative	506563.40	107	5.58/4.43	45.0/49.7	−1.36	−4.9	−5.17	1.02E–7	3	7%	156
		129	5.30/4.43	33.3/49.7	−1.01	−4.35	−3.99	1.53E–7	5	13%	242
		130	5.15/4.43	33.8/49.7	−1.31	−10.73	−9.26	4.56E–8	6	17%	438
		171	4.71/4.43	28.6/49.7	1.8	1.69	2.07	1.49E–8	7	22%	73
		173	4.65/4.43	25.5/49.7	2.53	6.33	5.29	8.44E–9	4	11%	237
		174	4.58/4.43	25.5/49.7	1.16	2.59	2.43	1.17E–6	4	11%	290
		176	4.47/4.43	25.0/49.7	1.7	3.58	3.52	2.60E–7	3	9%	216
		183	4.34/4.43	19.4/49.7	1.15	2.38	1.93	1.27E–5	2	6%	101
		184	4.25/4.43	19.4/49.7	1.56	5.97	5.14	3.49E–8	2	6%	116
		185	4.25/4.43	21.2/49.7	1.67	4.39	4.22	3.61E–6	2	6%	133
Calreticulin, putative	510685.10	81	6.37/4.49	42.2/46.2	−1.07	1	1.52	1.81E–5	2	6%	89
Chaperonin containing t-complex protein, putative	511725.250	12	5.02/4.80	69.7/59.2	−1.95	−12.81	−16.4	4.12E–7	3	9%	113
Chaperonin HSP60; mitochondrial precursor; GroEL protein; heat shock protein 60 (HSP60)	507641.290 or 507641.300 or 510187.551	17	5.44/5.14	66.2/59.2	−4.9	−19.19	−19.86	1.27E–8	9	24%	470
		18	5.55/5.14	66.0/59.2	−4.07	−9.24	−10.18	1.75E–8	3	20%	111
		20	5.65/5.14	65.8/59.2	−3.99	−8.83	−9.9	1.25E–7	7	18%	354
		23	5.22/5.14	62.3/59.2	−2.03	−8.02	−9.51	6.42E–8	7	18%	191
		24	5.13/5.14	62.2/59.2	−1.72	−11.52	−13.96	9.42E–8	11	31%	533
		25	5.21/5.14	61.5/59.2	−1.78	−16.99	−15.1	1.61E–7	6	16%	205
		28	5.29/5.14	62.0/59.2	−2.16	−9.12	−9.25	3.25E–8	5	14%	195
		88	4.68/5.14	51.2/59.2	−1.06	−3.44	−2.79	9.76E–7	3	18%	112
		89	4.83/5.14	51.8/59.2	−1.28	−7.85	−7.58	3.21E–7	3	18%	150
		106	5.5/5.14	43.1/59.2	1.23	−5.57	−4.86	2.11E–7	6	15%	177
		131	5.03/5.14	34.0/59.2	−1.76	−9.8	−7.3	3.45E–8	1	5%	76
		162	5.66/5.14	20.3/59.2	1.97	1.81	1.97	4.31E–7	2	10%	62
Chaperonin; Tcomplex-protein 1; theta subunit; putative	506247.50	16	5.42/5.12	68.9/58.3	−2.42	−4.81	−5.44	6.21E–8	3	7%	138
Cystathionine beta-synthase, cysteine synthase, serine sulfhydrylase (CBS)	508177.120 or 506905.50 or	78	6.83/7.14	45.0/47.0	−1.1	−2.96	−2.21	1.61E–7	4	13%	206
	508175.360 or 511691.10	80	6.37/7.14	45.3/47.0	−1.98	−10.23	−12.17	1.75E–8	2	7%	88
Cytochrome c oxidase subunit IV; putative	506529.360 or 510889.50	124	5.51/5.96	36.0/38.9	−1.01	−1.92	−1.58	5.98E–7	2	7%	105
Cytochrome c oxidase subunit V, putative	510565.30 or 508503.20	200	5.5/6.4	14.8/22.2	1.99	6.2	5.12	1.53E–7	2	14%	69
D-isomer specific2-hydroxyacid dehydrogenase-protein	510099.120	119	6.72/6.41	35.2/38.5	−1.46	−6.4	−5.31	6.69E–7	12	36%	103
		197	5.26/6.41	11.2/38.5	1.13	−4.38	−3.75	9.76E–9	6	20%	321
Dihydrolipoamide acetyltransferase precursor	509717.20 and 510105.170	0	5.75/6.39	62.2/49.6	−1.32	−7.65	−5.03	1.75E–8	1	3%	61
		33	5.91/6.68	62.1/49.6	−1.49	−7.45	−6.79	4.65E–9	4	14%	167
Dihydrolipoyl dehydrogenase; putative (GCVL-2)	507089.270 or 511025.110	73	6.69/7.4	52.5/54.9	1.01	−2.41	−2.34	2.36E–7	3	7%	98
Dipeptidyl-peptidase	508601.141 or 509205.120	29	5.40/5.60	62.4/74.4	−2.19	−5.73	−4.96	5.14E–8	2	3%	58
		30	5.48/5.63	62.3/74.4	−1.9	−7.12	−8.88	1.75E–8	3	6%	139
Drug resistance protein	444777.10	123	5.54/4.05	37.2/50.3	−1.18	1.68	2.34	5.72E–6	1	5%	26
Elongation factor 2, putative	510963.90	36	6.36/5.86	55.8/94.2	−1.82	−6.16	−5.58	5.78E–8	4	6%	197
		49	5.95/5.86	54.6/94.2	−1.99	−7.15	−6.91	2.60E–7	3	4%	130
		50	5.99/5.86	54.6/94.2	−1.93	−11.21	−11.53	3.72E–9	4	6%	146
		65	5.78/5.86	50.2/94.2	−1.4	−12.96	−9.24	3.48E–7	6	8%	325
		125	5.34/5.86	36.0/94.2	−1.2	−7.53	−7.72	1.34E–9	5	9%	293
		112	5.96/5.86	38.0/94.2	1.11	−2.38	−1.3	6.35E–4	4	6%	151
		137	5.38/5.86	30.7/94.2	1.83	1.32	1.98	2.44E–7	3	2%	92
Enolase	504105.140	72	6.54/6.2	50.6/46.4	−2.63	−5.28	−6.37	6.94E–8	2	7%	73
Eukaryotic translation initiation factor 6 (elF-6); putative	506679.70	168	5.04/6.09	20.7/33.2	1.55	1.31	1.27	3.90E–5	2	9%	127
Glucose-regulated protein 78, putative	506585.40	2	5.19/4.82	76.9/71.3	−2.45	−23.64	−16.54	1.02E–7	4	12%	198
		13	4.98/4.82	67.1/71.3	1.21	−2.77	−2.68	1.91E–6	2	4%	88
		95	4.72/4.82	45.7/71.3	−1.08	−4.09	−3.33	7.81E–7	2	3%	74
		96	4.58/4.82	45.4/71.3	1.52	1.68	1.52	4.25E–5	4	9%	297
Glutamamyl caboxypeptidase; putative	507689.40 or 507657.20 or 507657.10	70	6.18/6.51	47.6/43.4	−1.22	−2.15	−2.41	1.66E–6	2	6%	92
		76	6.53/6.51	47.0/43.4	−1.28	−2.09	−2.19	5.13E–7	2	6%	110
		77	6.59/6.51	45.5/43.4	−1.23	−2.99	−2.31	1.36E–7	3	9%	129
Glutamate dehydrogenase	508111.30	212	6.72/8.05	15.9/45.0	1.62	1.64	2	6.63E–6	2	7%	77
		213	6.79/8.05	15.9/45/0	1.88	2.39	2.64	4.83E–8	2	7%	110
		214	6.78/8.05	15.1/45.0	1.86	3.66	4.03	6.54E–9	4	13%	173
Glycerate kinase, putative	508741.170	159	6.49/8.21	20.7/56.1	1.37	−1.87	−2.19	1.43E–6	1	3%	37
Heat-shock protein 70kDa, putative	509543.50 and 511257.10	1	5.14/4.55	76.1/70.0	−2.15	−9.69	−10.51	3.28E–8	2	11%	70
		90	4.90/4.55	51.8/70.0	−1.16	−4.64	−5.4	2.91E–7	2	11%	79
		91	4.98/4.60	52.6/70.0	−1.12	−4.52	−3.7	2.44E–7	4	18%	205
Heat-shock protein 70kDa, putative	506135.9	155	5.63/6.56	23.4/30.2	1.7	2.79	2.2	5.79E–5	2	10%	88
Heat-shock protein 70kDa, putative	511211.160	7	5.55/5.85	72.7/70.9	−1.34	−4.53	−3.34	1.15E–7	4	15%	191
		92	5.00/5.85	47.8/70.9	−1.11	−5.67	−5.23	8.47E–8	2	11%	67
		101	5.13/5.85	44.1/70.9	1.3	−3.49	−3.28	4.17E–9	2	4%	67
		105	5.50/5.85	44.9/70.9	−1.49	−4.58	−3.7	1.75E–7	2	4%	78
		156	5.86/5.85	23.1/70.9	1.87	2.21	1.66	2.44E–7	4	19%	181
		160	6.72/5.85	23.0/70.9	1.08	−2.66	−2.99	2.96E–8	13	48%	107
		164	5.32/5.85	22.1/70.9	1.65	1.87	1.5	8.47E7	4	14%	214
		175	4.56/5.85	24.1/70.9	1.23	1.34	1.38	8.68E–5	1	2%	66
		177	4.65/5.85	20.8/70.9	2.01	5.22	4.11	5.33E−8	1	2%	72
Heat shock 70 kDa protein, mitochondrial precursor, putative	507029.30	8	5.65/5.71	72.4/71.0	−1.39	−4.87	−3.73	9.36E–9	2	4%	81
		9	5.77/5.71	72.7/71.0	−1.42	−5.3	−4	9.88E–9	6	14%	293
		10	5.90/5.71	73.1/71.0	−1.32	−3.83	−3.08	7.83E–8	3	7%	85
		11	5.87/5.71	69.7/71.0	−1.05	−1.87	−1.94	1.55E–5	4	10%	152
		19	5.60/5.71	67.4/71.0	1.18	−1.4	−1.53	1.17E–6	5	12%	285
		21	5.73/5.71	67.7/71.0	1.26	−1.42	−1.58	1.70E–7	4	10%	109
Heat-shock protein 85kDa, putative	509643.130 or 507713.30 or 509105.140	93	4.89/4.79	47.6/80.7	−1.44	−5.4	−4.32	7.81E–7	2	3%	87
		97	4.90/4.79	43.2/80.7	1.43	−1.5	−1.15	3.25E–7	3	5%	114
		98	4.95/4.79	43.0/80.7	1.02	−3.01	−3.37	1.49E–8	2	3%	145
		104	5.30/4.79	41.5/80.7	−1.63	−16.15	−18.55	2.26E–8	3	5%	143
		126	5.40/4.79	33.5/80.7	1.2	−3.77	−3.74	4.34E–8	2	3%	89
		134	4.62/4.79	40.6/80.7	1.39	2.58	2.18	7.35E–6	4	6%	258
		148	4.95/4.79	28.8/80.7	1.76	2.78	2.47	3.49E–8	1	1%	100
		149	4.86/4.79	28.9/80.7	1.67	3.55	3.23	6.89E–8	1	1%	62
Hypothetical protein, conserved	505989.110	182	4.46/4.50	18.4/22.2	−1.25	3.47	3.51	1.92E–5	2	11%	62
Hypothetical protein, conserved	506605.120 or 511239.110	202	5.72/4.99	15.1/28.6	2.75	7.08	7.83	1.34E–9	4	22%	174
		203	5.67/4.99	14.2/28.6	1.87	3.96	3.33	3.66E–6	9	41%	92
		204	5.86/4.99	14.1/28.6	1.48	2.12	2.78	1.88E–7	6	31%	326
Hypothetical protein	508817.20 or 503801.70	154	5.38/8.58	23.5/66.7	1.78	4.80	4.29	3.96E–8	1	1%	17
Nucleoside phosphorylase, putative	508989.9 and 509569.100	121	6.90/6.42	34.2/37.0	−1.18	−5.59	−4.38	2.96E–8	3	16%	190
		118	6.38/6.42	35.6/37.0	−1.12	−3.17	−2.56	1.17E–8	1	4%	23
Oligopeptidase B, putative	503995.50	47	5.86/6.1	55.2/80.8	−1.71	−5.94	−7.55	9.76E–9	2	3%	68
		63	5.86/6.1	52.0/80.9	−1.12	−2.26	−2.47	8.01E–7	2	3%	69
Paraflagellar rod protein 3	509617.20	60	6.09/5.96	56.1/68.6	1.21	−3.51	−4.33	6.89E–8	1	2%	24
Peptidase M20/M25/M40	510257.80	39	5.57/5.19	55.4/51.2	−1.74	−5.55	−7.07	8.47E–7	2	6%	66
		40	5.50/5.19	54.4/51.2	−1.64	−5.35	−9.41	2.26E–8	1	3%	31
Peroxiredoxin; tryparedoxin peroxidase	509499.14	189	5.16/7.92	18.1/25.5	1.11	1.42	1.73	7.81E–7	3	15%	174
Phosphoglycerate kinase, putative or 3-phosphoglycerate kinase, glycosomal (PGKA)	511419.40 or 505999.90 or 511419.50 or 505999.100	74	6.76/7.4	51.9/54.90	−2.99	−5.56	−5.35	2.46E–7	1	3%	78
Prostaglandin F2 alpha synthase (TcPGFS)	508461.80	14	5.11/6.43	68.1/42.2	−1.19	−6.45	−7.01	1.59E–6	4	14%	169
		111	5.91/6.43	40.0/42.2	−1.31	−11.76	−7.75	1.74E–8	5	17%	284
		113	6.10/6.43	40.0/42.2	−1.11	−10.19	−6.05	1.75E–8	8	38%	116
		114	6.09/6.43	38.7/42.2	1.12	−6.24	−5.06	3.12E–7	4	13%	188
		144	6.15/6.43	26.7/42.2	1.49	−1.65	−1.39	5.49E–8	4	13%	217
		161	5.52/6.43	22.1/42.2	1.74	1.88	1.99	6.34E–8	9	31%	92
Protein disulfide isomerase	506247.10 or 507611.370	180	4.42/4.6	20.9/53.5	1.46	3.7	3.6	2.44E–7	2	4%	44
Pyruvate dehydrogenase E1 beta subunit; putative	510091.80	132	5.03/5.02	30.9/37.8	1.08	−2.21	−1.88	1.27E–6	5	20%	191
		133	4.62/5.02	40.7/37.8	1.02	−4.58	−3.83	4.13E–7	2	6%	108
Pyruvate kinase 2, putative	507993.390 or 511281.60	68	5.97/7.44	46.6/54.6	−1.3	−4.94	−4.8	1.73E–7	1	2%	29
Pyruvate phosphate dikinase	510101.140	194	5.00/8.27	13.5/100.8	1.83	10.51	7.42	7.88E–9	2	3%	94
Receptor for activated C kinase 1, putative	511211.120 or 511211.130	122	5.93/6.04	35.4/35.0	−1.22	−6.66	−6.06	2.31E–9	3	11%	122
S-adenosylhomocysteine hydrolase	511229.50 or 511589.200	193	5.25/6.64	12.9/48.4	1.41	−1.23	−1.1	3.74E–6	2	7%	80
Seryl-tRNA synthetase	511163.1 or 506777.80	140	5.38/5.41	28.9/25.7	1.4	−1.07	1.28	3.36E–6	3	19%	97
succinyl-CoA ligase [GDP-forming] beta-chain, putative	507767.10	150	4.92/5.58	26.2/34.5	1.63	2.03	2.25	1.08E–6	2	7%	87
Thiol−dependent reductase 1; putative; thiol transferase; putative; glutathione s-transferase; putative	509105.70 or 503419.30	62	6.00/5.83	51.9/50.7	−1.18	−2.07	−1.56	4.56E–6	2	6%	86
		158	6.21/5.83	21.5/50.7	−1.32	−2.17	−2.64	4.48E–6	1	3%	32
Trans-sialidase	509927.10	186	4.18/6.67	21.0/54.7	1.67	4.7	4.71	6.10E–9	1	4%	25
Tryparedoxin peroxidase	487507.10 or 509445.10 or 504839.28 or 507259.10	210	6.24/6.75	11.5/22/4	2.17	3.18	3.42	8.05E–5	1	5%	33
		211	6.66/6.75	17.0/22.4	−1.52	−3.96	−1.82	3.25E–8	3	22%	105
		215	6.75/6.75	11.8/22.4	1.44	−1.3	1.25	1.83E–6	3	19%	109
Tyrosine aminotransferase	510187.20 and 510187.50 or 510187.40 or 510187.30	64	5.83/7.2	50.6/46.1	−1.22	−1.77	−1.51	5.02E–5	1	2%	58
		79	6.25/6.14	44.9/46.1	−2.36	−31.03	−28.73	1.34E–9	4	16%	160
		115	6.21/6.14	38.9/46.1	−1.05	−7.27	−7.38	9.64E–9	4	11%	110
		116	6.18/6.14	37.0/46.1	1.09	−5.43	−4.73	8.44E–9	2	14%	128
Vacuolar ATP synthase subunit B	506025.50 or 511209.10	37	5.71/5.29	59.1/55.5	−1.69	−3.54	−4.01	2.44E–7	6	21%	207

**Figure 3:**
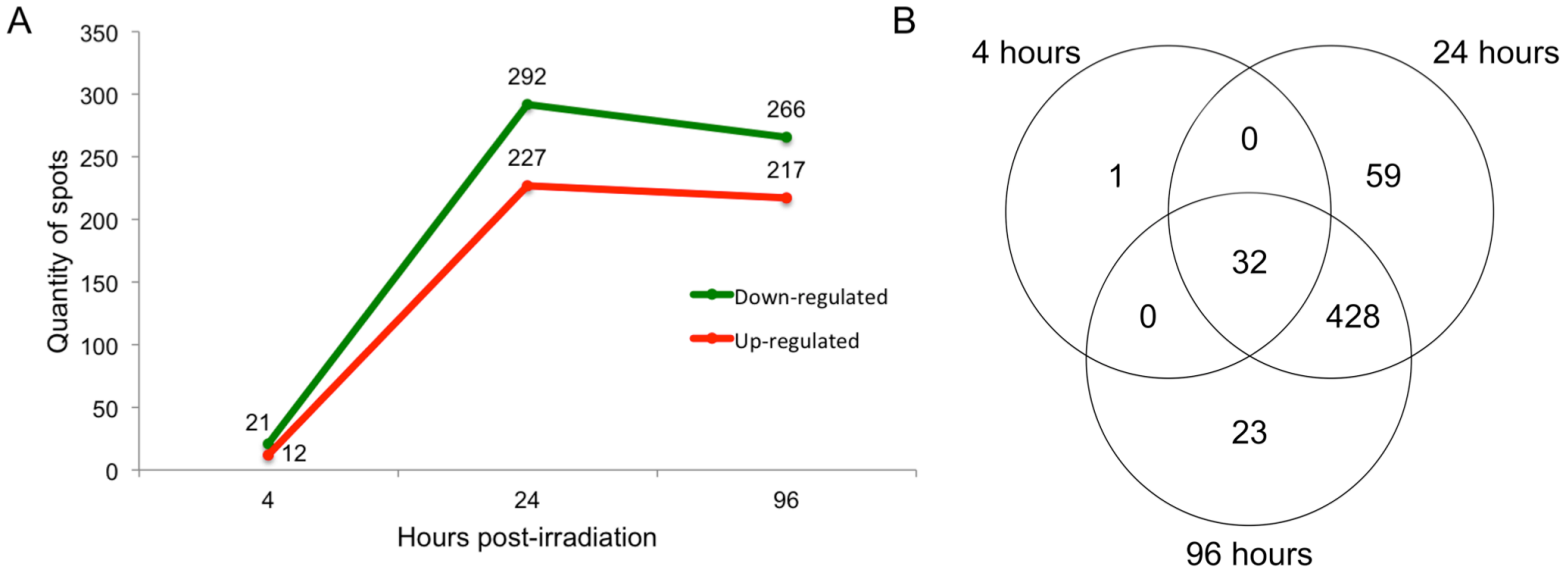
Protein spots differentially expressed at all time points. A) Number of downregulated and upregulated protein spots per time point. B) Venn diagram showing the overlaps of 32 protein spots differentially regulated among the three time points and of the 428 protein spots between 24 and 96 hours.

Additionally, the Student's t-test (p<0.01) was applied to verify which proteins were differentially expressed in each time point when compared with the NI sample. The overall and time-specific number of downregulated protein spots was higher than the number of upregulated ones (Figure 3A). These findings are different from those described previously in our microarray study. Twenty-four hours post-irradiation, the number of downregulated genes decreases drastically, reaching only 6 down-expressed transcripts 96 hours after irradiation, while the number of upregulated genes increases [7]. A linear regression analysis between mRNA and protein levels from genes concomitantly identified in both studies was carried out for each time point. The correlation was extremely poor at all time points, starting with multiple R2 = 0.064 at 4 hours and reaching R2 = 0.27 and 0.24 at 24 and 96 hours post-irradiation, respectively (data not shown). Although a very low correlation was obtained, the result is in agreement with other studies performed in both prokaryotes and eukaryotes using classical methodologies such as microarray, Serial Analysis of Gene Expression (SAGE) and RNA-Seq for transcriptomic expression data, and 2-DE, Multi-dimensional protein identification technology, and MS for proteomics data [26]–[31]. This analysis reinforces the idea that transcriptomic and proteomic approaches are complementary, not confirmatory [29].

Moreover, changes in the *T. cruzi* proteome are more evident 24 hours after exposure to gamma radiation (Figure 3). This scenario suggests that epimastigote cells present an immediate but subtle response to gamma radiation characterized by 12 induced and 21 repressed protein spots 4 hours after irradiation (Figure 3A). Between 4 and 24 hours after irradiation, a more intense response to stress was observed and most of the induced and repressed protein spots were still significantly altered until 96 hours (428 spots; Figure 3B). This finding indicates a sustained alteration in the abundance of specific *T. cruzi* proteins 24 hours after gamma radiation exposure. When analyzing the *T. cruzi* proteome 24 hours after irradiation, we found that, from the 59 exclusive spots, approximately 66% were repressed and 34% were induced. However, the majority of the 23 exclusive spots found 96 hours after irradiation were induced (approximately 61%).

### Exposure to Gamma Rays Increases the Levels of Shorter and/or Processed Proteins in Epimastigote Cells

**Figure 4:**
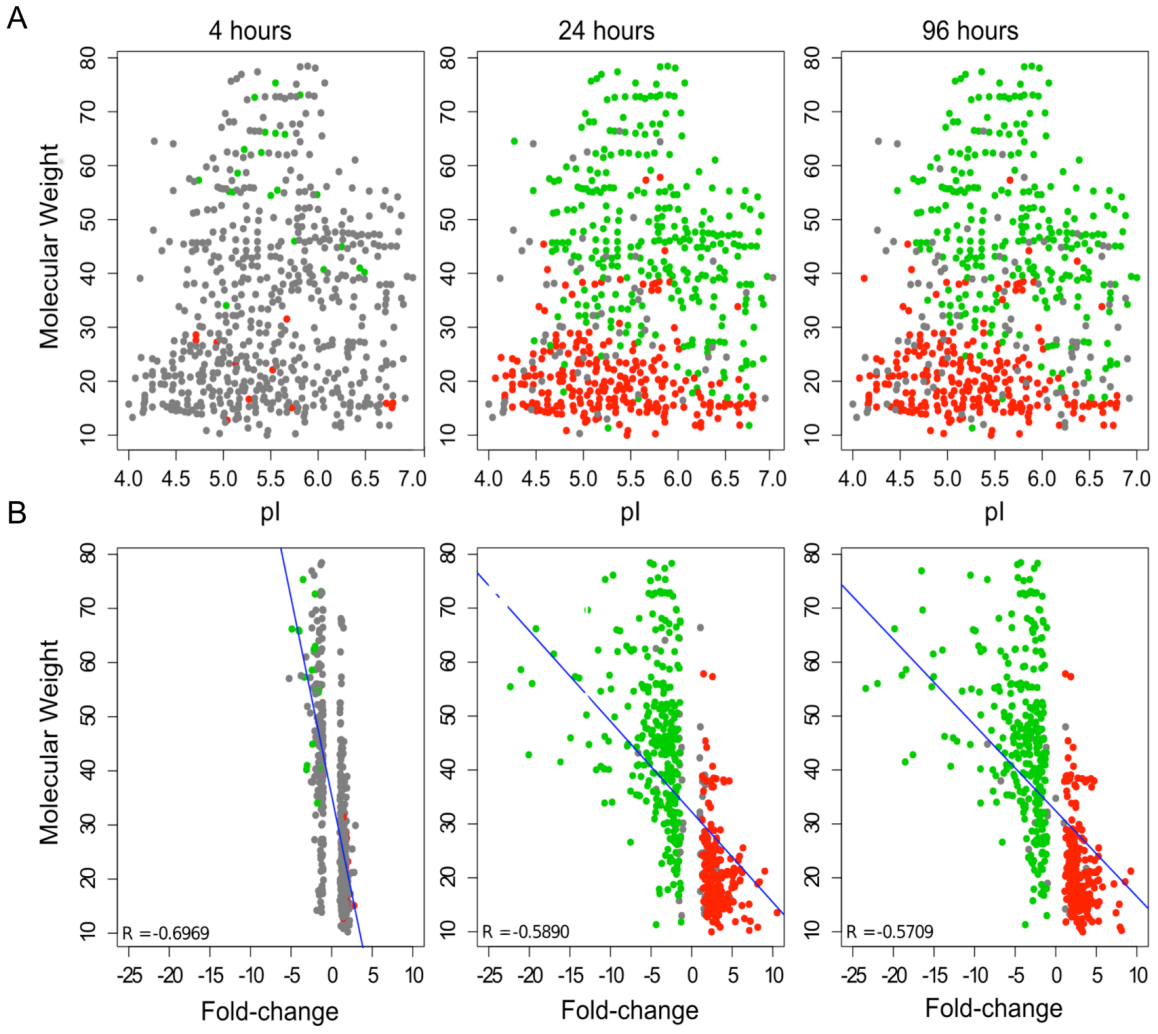
Distribution of upregulated and downregulated protein spots versus molecular weight, pI, and fold change. In the scatter plots, upregulated protein spots are shown in red and downregulated protein spots are shown in green. The correlation between molecular weight and pI or fold-change ratio is shown in (A) and (B), respectively. Spots with no significant difference in expression are colored gray. The blue line indicates the negative correlation between molecular weight and fold change.

**Figure 5:**
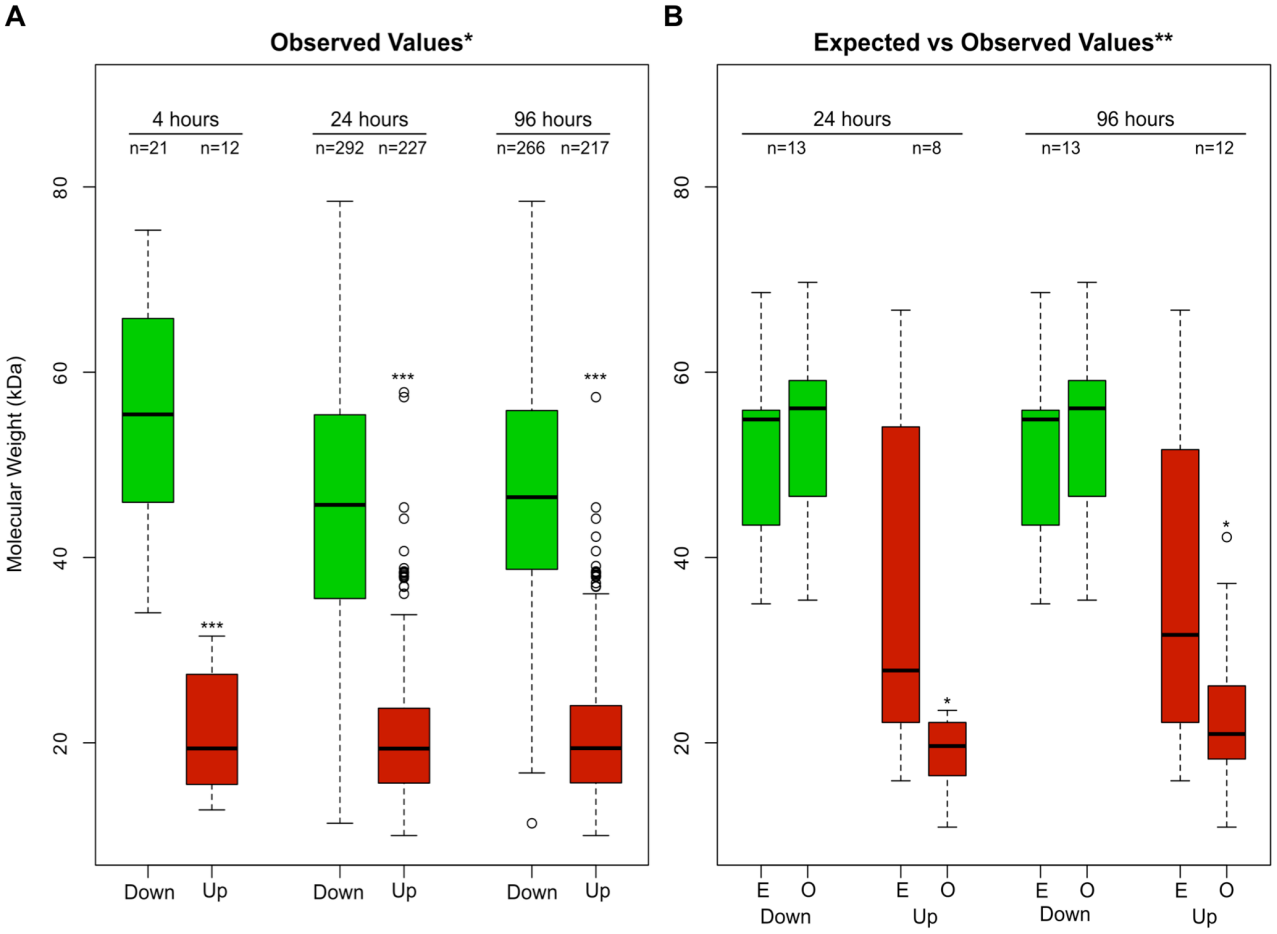
Boxplots of peptide molecular weights. A) Distribution of the observed molecular weight in downregulated (green) or upregulated (red) protein spots at each time point analyzed. B) comparison between the distribution of the expected (E) and observed (O) molecular weights among downregulated or upregulated protein spots 24 and 96 hours after irradiation. A single asterisk corresponds to p<0.05 and a double asterisk corresponds to p<0.001.

When analyzing the set of upregulated proteins (especially 24 and 96 hours after irradiation), we observed a tendency for the overexpression of shorter molecules to the detriment of longer ones. The upregulated protein spots (red-colored dots) are mainly at the lower part of the gel (lower molecular weight), while the downregulated protein spots (green-colored dots) are more sparsely distributed across the gel (Figure 4A). In addition, low molecular weight protein spots tended to have larger fold changes when compared with those with molecular weights close to the expected value (Figure 4B). The Wilcoxon test was applied and confirmed that the median values of the molecular weight of downregulated and upregulated protein spots were different for each time point (p<1e-09; median values of 55.45/19.39, 45.64/19.38, and 46.51/19.42 for 4, 24, and 96 hours, respectively; Figure 5A).

When we considered the expected molecular weight of the full-length isoform (predicted size obtained from the TriTryDB website) of both upregulated and downregulated proteins, we noticed a decrease in protein size in the former case and an increase in the latter case, thus showing the emergence of lower molecular weight protein isoforms after irradiation (Figure 5B). We then decided to confirm if the observed molecular weight of these proteins in the 2D gels was in agreement with their expected molecular weight. In the case of upregulated proteins, the observed molecular weight was significantly lower than expected (Figure 5B), indicating that these proteins might be processed, yielding shorter polypeptides. It is important to note that this result does not seem to be a consequence of protein degradation, since clear spots can be observed in the 2D gel, indicating the presence of a large amount of identical polypeptides in this region of the gel. This would not be the case if proteins were degraded, considering that in this situation peptides of variable size would be generated and no clear spot would be observed in the gel. These results may indicate the emergence of new protein isoforms, as the result of protein processing, alternative splicing of mRNAs, and/or alternative translational start/stop sites after irradiation. Alternative splicing of transcripts has the potential to expand the repertoire of proteins. Recent studies have estimated that all multi-exonic human genes are able to produce at least two alternatively spliced mRNA transcripts by alternative splicing, generating different proteins isoforms with altered structures and biological functions [32]. In trypanosomatids, mature mRNAs are generated after two processing events: trans-splicing to add the spliced leader (SL) sequence to the 5′ end of transcripts and subsequent polyadenylation [17]. A genome-wide analysis comparing the SL addition site along the developmental cycle of the parasite suggests that alternative trans-splicing plays an important role in differential gene expression [33]. The occurrence of alternative trans-splicing could be an explanation for the presence of so many different isoforms in *T. cruzi* after radiation response.

A similar event has already been described in *D. radiodurans*, since different isoforms of the single-strand binding (SSB) protein were produced after ionizing radiation stress induction. SSB proteins are vital for cell survival due to their involvement in processes such as DNA replication, recombination, and repair. The SSB protein spots in the gel followed a dynamic pattern of appearance, indicating a progressive processing of the C-terminal acidic tail, perhaps upon its interaction with ssDNA. The observed isoelectric point (pI) and molecular weight of deinococcal SSB isoforms were in agreement with the *in silico*-predicted pI and molecular weight of the SSB proteins shortened from the C-terminal end [16].

Intriguingly, in most of the observed processed proteins, the identified peptide sequences were the same or nearly the same in all sequenced protein spots and, therefore, it was impossible to define the actual outcome of the protein processing. As a particular case of study, the protein annotated as prostaglandin F2 alpha synthase, which is similar to NADH-flavinoxidoreductase, is processed to a total of six different forms (Figure S2). While the expected molecular weight for the annotated sequence is of 42 kDa, only two isoforms are nearly this size (∼40 kDa) and were, in fact, the most downregulated isoforms. A third isoform has a predicted molecular mass of 68 kDa, greatly exceeding the expected protein size. As all of the MS/MS-identified peptides were mapped to the C-terminal portion of the isoforms, there is no information to characterize the N-terminal of this enlarged protein naturally present in the NI parasites and downregulated after exposure to gamma radiation. A smaller (29 kDa) protein is expressed in approximately equal levels before and after radiation exposure, while an even smaller (22 kDa) protein species is exclusively present in irradiated cells. This is an interesting example, representative of multiple cases, in which we have observed the emergence of shorter isoforms of a same protein after epimastigote irradiation. The list of processed proteins expressing shorter isoforms after irradiation includes alpha and beta-tubulin, D-isomer specific 2-hydroxyacid dehydrogenase-protein, elongation factor 2, glycerate kinase, pyruvate dehydrogenase E1 beta subunit, tyrosine aminotransferase, and several heat shock proteins (HSPs), such as HSP60, DnaK, HSP70s, and glucose-regulated protein 78. Apart from the previously discussed SSB proteins in *D. radiodurans*, very few references in the scientific literature mention the presence of shorter protein fragments after radiation exposure in any organism.

Interestingly, Parodi-Talice and collaborators [34] observed a similar pattern in *T. cruzi* for the proteins glutamate dehydrogenase (GluDH), HSP70, and alpha and beta-tubulins, where lower molecular weight isoforms were differentially expressed during metacyclogenesis when compared with isoforms with the predicted molecular weight. The transformation of epimastigotes into metacyclic trypomastigotes is a complex process of differentiation, requiring a controlled production of various proteins [34]. Similarly, a quantitative time-course proteome analysis for the schizont-stage of *Plasmodium falciparum* (34 to 46 hours after invasion) demonstrated that actin-I, enolase, HSPs, and eukaryotic initiation factor 4A and 5A presented more than one isoform. The isoforms also showed different expression patterns at the different time points analyzed. *P. falciparum* is characterized by a complex life cycle, undergoing extensive morphological and metabolic changes, which reflects its capacity to survive in different host environments [35]. According to the authors, post-translational modifications may be a very important strategy for the parasites to control gene expression during differentiation [34], [35].

### Differentially Expressed Proteins after Gamma Radiation Exposure

Regarding the differentially expressed proteins, many of the listed proteins in Table 2 and Figure S3 are related to the protein synthesis process that seems to be upregulated, except for some protein spots of the elongation factor 2 that show a reduction in their levels. This may be a response to compensate for the processing of proteins that occurs after irradiation. This response may also enhance the synthesis of specific proteins that will possibly play a role in the stress response. The results obtained from the analyses of translation inhibition and proteomic profile after irradiation place *de novo* protein synthesis as an important cellular response to gamma radiation. The same pattern is observed in *D. radiodurans*, where proteins related to translation/folding displayed either enhanced or *de novo* expression in the first hour of post-irradiation recovery. Proteins involved with DNA repair and oxidative stress alleviation were also induced in *D. radiodurans* under ionizing radiation stress [16].

Proteins involved in protein folding processes, such as chaperones, are mostly downregulated post-irradiation (Figure S3). This represents an unexpected result, since these proteins are classically involved with stress response by stabilizing newly synthesized protein molecules. Nevertheless, this result is in agreement with transcriptomic data observed in microarray experiments [7]. It is worth noting that, although HSPs are mostly downregulated, processed forms of these molecules are upregulated and may even be functional. Interestingly, the two chaperones localized in the endoplasmic reticulum (calreticulin and protein disulfide isomerase) are upregulated after gamma radiation exposure, which may indicate an important role of this compartment in the ionizing radiation stress response, suggesting the existence of an unfolded protein response-like in this condition [36].

Another unexpected result is the downregulation of proteins involved in the ATP metabolism (namely the beta subunit of ATP synthase and the subunit IV of cytochrome c oxidase), although another member of this class is upregulated (cytochrome c oxidase subunit V). The outcome of this result is not clear and a more in-depth study of the cell energy metabolism would be important.

Perhaps the most remarkable observation in the post-irradiation proteome investigated here is the putative decline in the activity of the glycolytic and amino acid metabolism pathways. Several important enzymes of glycolysis were downregulated after gamma radiation exposure. Accordingly, the only enzyme (pyruvate phosphate dikinase) from gluconeogenesis listed here was upregulated. Most enzymes involved in the amino acid metabolism were also downregulated, but shorter isoforms of the GluDH were upregulated after irradiation. They consist of three isoforms with experimental molecular weights (15 kDa) lower than the predicted values (45 kDa), suggesting once again the occurrence of post-transcriptional modifications/processing of important metabolic enzymes during the stress response. GluDH catalyzes the NAD- and/or NADP-dependent reversible deamination of L-glutamate to form alpha-ketoglutarate and is essential for the metabolism of amino nitrogen in organisms ranging from bacteria to mammals [37]. *T. cruzi* has a metabolism that is largely based on the consumption of amino acids, mainly, proline, aspartate, and glutamate, which constitute the main carbon and energy sources of the epimastigote forms. In *T. cruzi*, GluDH has NADP-specific activity [38], indicating that it may serve as a pentose-phosphate shunt-independent source of NADPH in these parasites. Taken together, these results suggest that the parasite experiences an overall reduction on its energy metabolism as a consequence of its growth arrest after irradiation.

We have identified four proteins classified as redox sensors in this study. While two of these are downregulated (both oxidoreductases), the other two are upregulated and these are both tryparedoxins, which efficiently reduce hydrogen peroxide [39]. Throughout its life cycle, *T. cruzi* is exposed to various stresses in different environments: the invertebrate (triatomine bugs) and the vertebrate hosts. One of the most deleterious consequences of oxidative stress may be the formation of DNA lesions. Guanine is the most susceptible base to oxidation, due to its low redox potential, and the 7,8-dihydro-8-oxoguanine (8-oxoG) is the most common lesion. When 8-oxoG is inserted during DNA replication, it can generate double-strand breaks, which makes this lesion severely deleterious. Recently Aguiar *et al*., 2013, demonstrated that parasites overexpressin MutT are more resistant to the oxidative stress caused by hydrogen peroxide (H_2_O_2_) treatment. The MutT enzyme product, 8-oxod-GMP, can generate an oxidative stress signal, enabling the cells to overcome this stress. MutT hydrolyses 8-oxo-dGTP in the nucleotide pool, returning it to the monophosphate form so that it cannot be incorporated into DNA by polymerases. Parasites overexpressing heterologous MutT also increase the levels of cytosolic and mitochondrial peroxidases (TcCpx and TcMPx) after H_2_O_2_ treatment. Taking this into account and also that parasites subject to gamma radiation experience oxidative stress and increase the levels of some antioxidant enzymes not immediately after irradiation, but later after irradiation, we could suggest that *T. cruzi* does not respond directly to ROS production as a consequence of irradiation, but to 8-oxo-dGMP that is generated subsequently. The nucleotide 8-oxo-dGMP, or another secondary metabolite generated from this process, could be acting as a second messenger to the cell and indicating the presence of oxidative stress.

Recently, Krisko & Radman proposed a new paradigm when a cell is subject to ionizing radiation: the proteome rather than the genome is the primary target in radiation-induced cell death. This paradigm has been supported by several experimental evaluations showing that *D. radiodurans* has a way of protecting its proteins from oxidative damage [40]. Indeed, a strong correlation between intracellular Mn/Fe concentration ratios and bacterial resistance to radiation has been shown, in which the most resistant bacteria tolerates 300 times more Mn^2+^ and three times less Fe^2+^ than the most radiation-sensitive bacteria [15]. Manganese ions prevent the formation of iron-dependent ROS through the Fenton reaction, acting as chemical antioxidant protectors. Furthermore, measurements of protein carbonyl groups in *D. radiodurans* revealed that Mn^2+^ accumulation prevented protein oxidation; these results were also observed in other radioresistant bacteria [41]–[44].

Furthermore, the level of oxidative protein damage caused during irradiation controls the survival of many organisms (*Bdelloid rotifers*, a class of freshwater invertebrates, *Caenorhabditis elegans,* the bacteria *D. radiodurans,* and *Halobacterium salinarum*), which are extremely resistant to ionizing radiation [44]–[47]. The principal factor responsible for this extraordinary radioresistance is their great antioxidant protection of their cellular constituents, including those required for DSB repair, allowing them to recover from stress and continue reproduction [46].

An important finding of this study is the significant upregulation of three hypothetical proteins after gamma radiation. This may indicate a role for species-specific proteins in the response to stress after ionizing radiation exposure, since these most likely represent proteins with no homologues in other species. Similarly, in an initial *D. radiodurans* proteome study, hypothetical proteins were identified and further proved to be crucial for the response to radiation in this bacterial species [47].

Sghaier and collaborators have recently published a study on the amino acid composition of proteins from radiation-resistant bacteria [48]. The authors report that such proteins bear more small amino acids and fewer aromatic rings. We have also assessed the amino acid composition of *T. cruzi* proteins in a slightly different perspective. Amino acid counts were performed for upregulated *T. cruzi* proteins after gamma radiation exposure (we have considered as upregulated the proteins that were more abundant than in NI cells at least in one time point) and for the orthologues in *T. brucei* of *T. cruzi* upregulated proteins. In both cases, amino acid counts were normalized by the count performed in the set of all annotated proteins of the respective *Trypanosoma sp*. The hypothesis was that proteins with important roles after irradiation in *T. cruzi* would have an amino acid composition different than what is observed in the set of all *T. cruzi* proteins and in the respective orthologues in *T. brucei* (which is not radio-resistant).

When we compared *T. cruzi* proteins that were upregulated after radiation exposure with the entire set of annotated proteins from this parasite, we observed that the former have in general fewer polar, hydrophobic, and small amino acids (although some amino acids in these classes are more frequent). In addition, upregulated proteins have fewer aromatic amino acids (except for tyrosine, which is more frequent) and less sulfur-containing cysteine residues.

## Conclusions

Using 2D-DIGE and MS, we have identified 543 protein spots differentially expressed after gamma radiation exposure. The presence of multiple isoforms was observed for more than half of the identified proteins, most of which are shorter than the annotated protein size in the *T. cruzi* genome. Additionally, there was a strong correlation for lower molecular weight peptide spots to be overexpressed. This result could be explained by *de novo* protein synthesis of different isoforms, protein processing, and/or modification events subsequent to radiation exposure. This observation indicates that post-translational control of gene expression have an important role in the parasite response to gamma radiation stress. The inhibition of protein synthesis in face of gamma radiation was shown to have a significant effect decreasing parasite growth and survival rates, highlighting the importance of active translation for parasite recovery after exposure to ionizing radiation.

We have annotated all 53 proteins identified by MS according to their biological roles. Several proteins were represented by multiple spots, and most of them had molecular weights lower than predicted. As a consequence of this observation, we cannot precisely state which biological processes are upregulated *versus* downregulated, since the different protein isoforms may not function in the same way as the full-length protein. Nevertheless, some tendencies could be observed in this study, including changes in the following biological processes: upregulation of the protein synthesis process, downregulation of protein folding (except for the upregulation of two endoplasmic reticulum chaperones), downregulation of the ATP generation pathway, glycolysis, and amino acid metabolism, and the upregulation of two tryparedoxins (which reduce hydrogen peroxide in response to oxidative stress).

Finally, taking into account the translation inhibition results obtained herein, together with the observed proteomic profile after irradiation, we can conclude that *de novo* protein synthesis is an essential cellular response to gamma radiation.

## Supporting Information

Figure S1 Electrophoretic analysis of total protein extracts of irradiated and NI epimastigote cells. Total protein extracts were obtained for each time point NI, 4, 24, and 96 hours after irradiation. Samples were subjected to 12% SDS-PAGE and stained with coomassie blue.
(TIF)

Figure S2 Differentially expressed isoforms of prostaglandin F2 alpha synthase. The upregulated protein spot (161) shows a lower molecular weight when compared with the downregulated proteins spots (14, 111, 113, 114, and 144).
(TIF)

Figure S3 Time point expression of protein spots.
(PDF)
