# Supplementary figures and images for: Proteomic Analysis of Trypanosoma cruzi Response to Ionizing Radiation Stress
Source: PLoS One. 2014 May 19;9(5):e97526. doi: 10.1371/journal.pone.0097526 (PMC4026238; doi:10.1371/journal.pone.0097526)

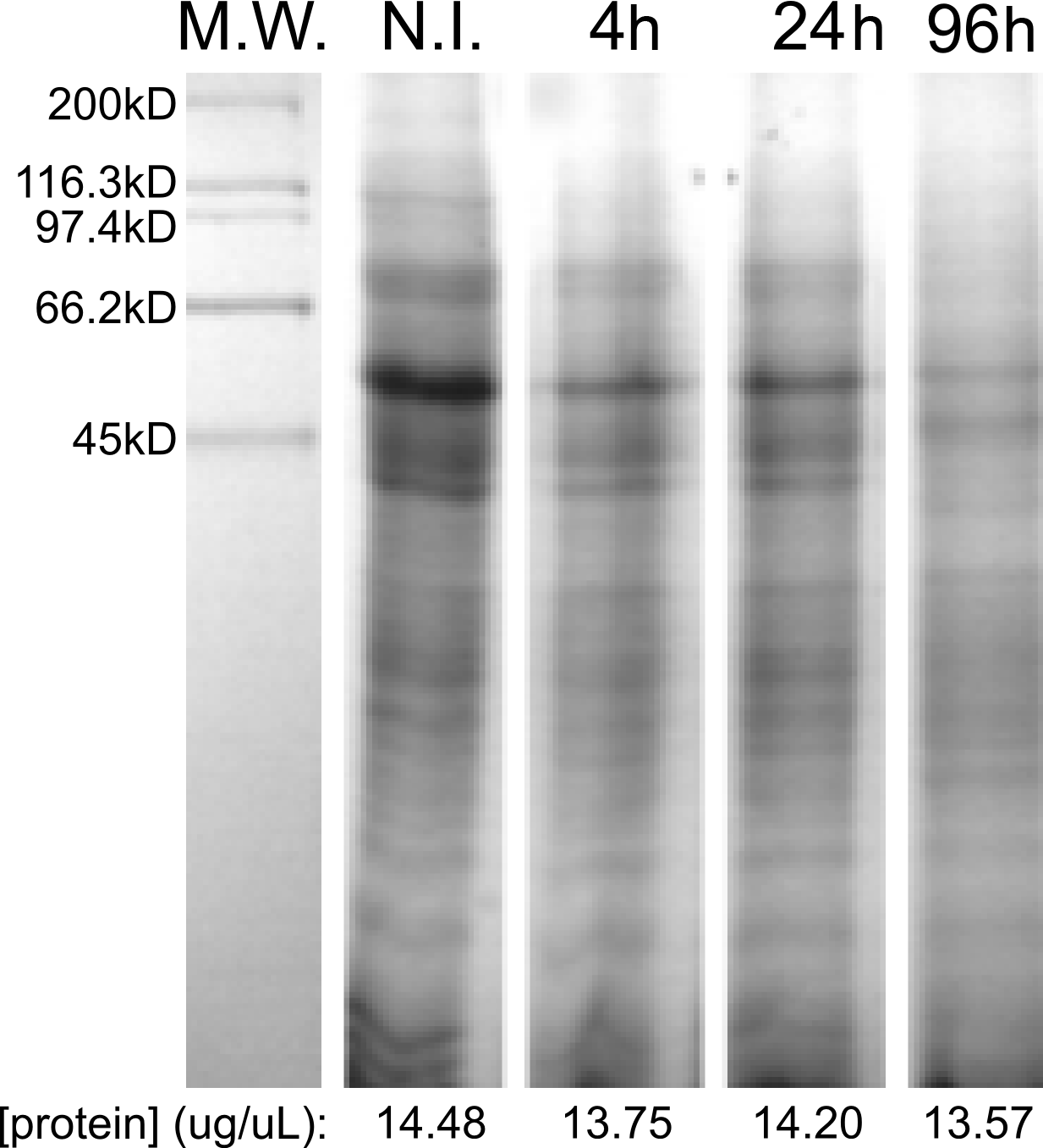

Supplement: Figure S1 — Electrophoretic analysis of total protein extracts of irradiated and NI epimastigote cells. Total protein extracts were obtained for each time point NI, 4, 24, and 96 hours after irradiation. Samples were subjected to 12% SDS-PAGE and stained with coomassie blue. (TIF) [file pone.0097526.s001.tif]
