# Supplementary material for: Proteomic Analysis of Trypanosoma cruzi Response to Ionizing Radiation Stress
Source: PLoS One. 2014 May 19;9(5):e97526. doi: 10.1371/journal.pone.0097526 (PMC4026238; doi:10.1371/journal.pone.0097526)
Supplement: Figure S3 — Time point expression of protein spots. (PDF) [file pone.0097526.s003.pdf]

TIME-POINT EXPRESSION OF PROTEIN SPOTS

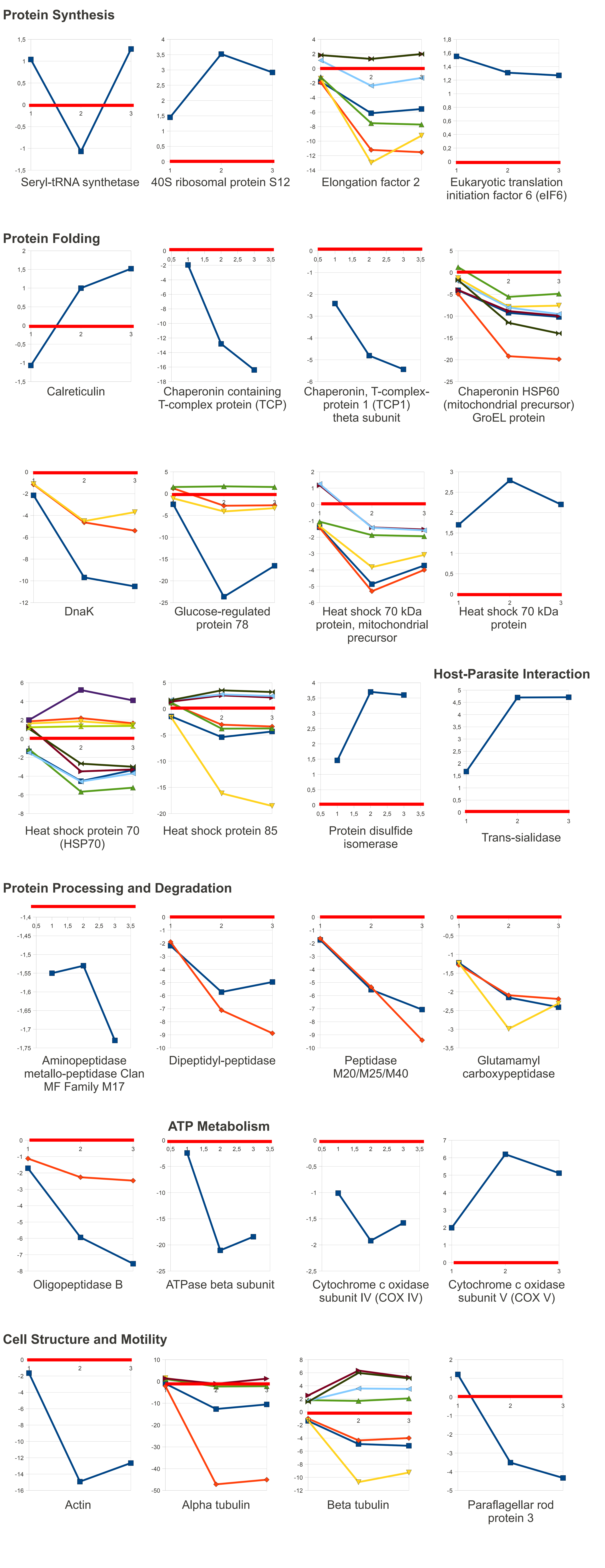

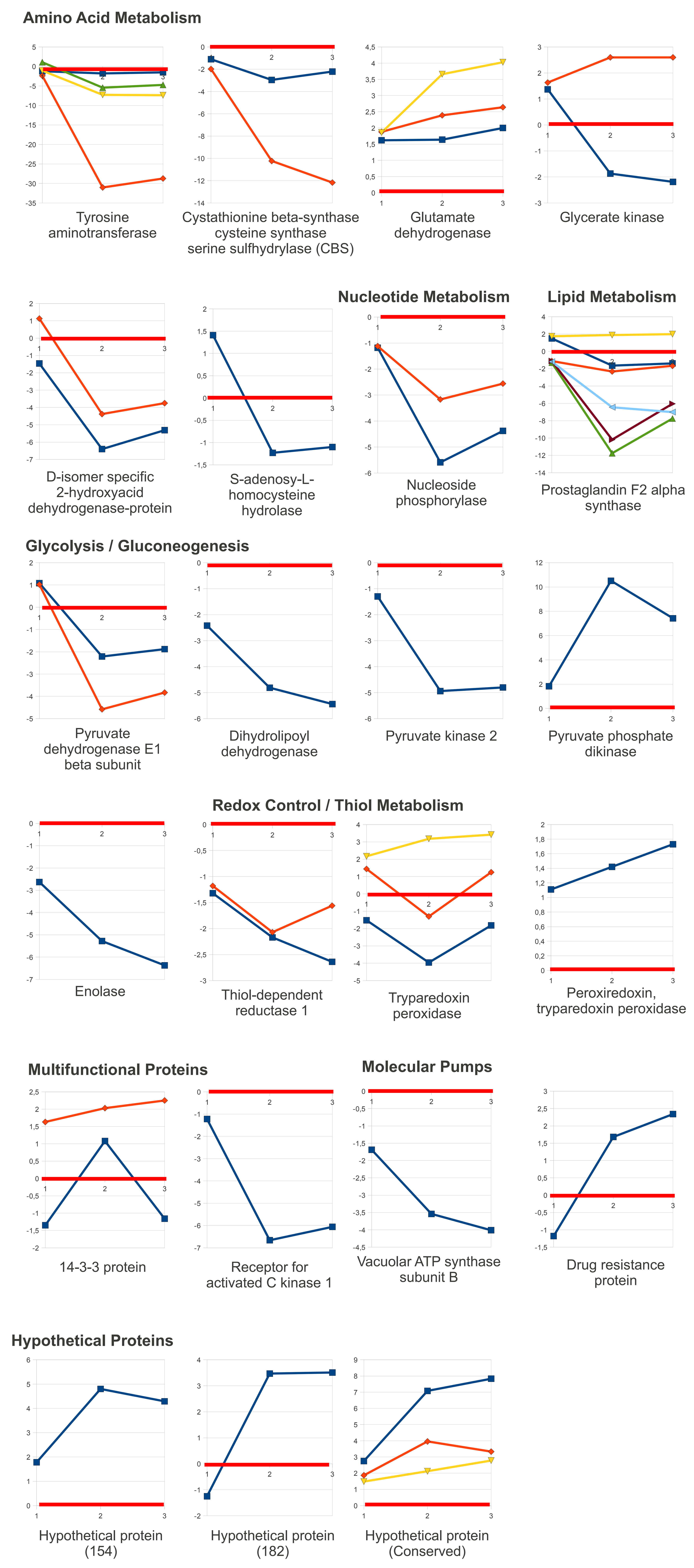

Expression is given according to fold change. The x-axis represents 4h (1), 24h (2) and 96h (3) and the y-axis represents the fold-change. Each spot of multispot proteins is represented by a different line color and the red line in all graphs represents the reference (the non-irradiated state, in which the fold-change is set to zero).
